# The Linear Hygroscopic Expansion Coefficient of Cement-Based Materials and Its Determination

**DOI:** 10.3390/ma13010037

**Published:** 2019-12-20

**Authors:** Depeng Chen, Qilin Zhu, Zhifang Zong, Tengfei Xiang, Chunlin Liu

**Affiliations:** 1 School of Architectural and Civil Engineering, Anhui University of Technology, Ma’anshan 243032, China; dpchen@ahut.edu.cn (D.C.); 15755568760@163.com (Q.Z.); zhifangzong@126.com (Z.Z.); xiangtf@ahut.edu.cn (T.X.); 2 Institute of Green Building Materials, Anhui University of Technology, Ma’anshan 243032, China; 3 School of Materials Science and Engineering, Southeast University, Nanjing 211189, China

**Keywords:** linear coefficient of hygroscopic expansion (LCHE), moisture-induced deformation, inversion analysis, cement-based materials, concrete

## Abstract

A crack caused by shrinkage could remarkably increase the permeability, heavily deteriorate the durability, and heavily deteriorate the service life of a concrete structure. However, different forms of thermal shrinkage can be predicted by directly applying a temperature load on a node. The prediction of moisture-induced stresses in cement-based materials by using the common finite element method (FEM) software is a big challenge. In this paper, we present a simple numerical calculation approach by using the proposed coefficient of hygroscopic expansion (CHE) to predict the moisture-induced deformation of concrete. The theoretical calculation formula of the linear CHE (LCHE) of cement-based material was deduced based on the Kelvin–Laplace equation and the Mackenzie equation. The hygroscopic deformation of cement mortar was investigated by inversion analysis; based on the results, the LCHE could be determined. Moreover, a case analysis of the application of LCHE to concrete is also conducted. The simulated results of concrete shrinkage were close to the experimental ones. As a whole, it is feasible to predict the drying shrinkage of concrete through simple calculation by using the proposed LCHE, which is also beneficial to the direct application of moisture loads on nodes in finite element analysis (FEA).

## 1. Introduction

The durability of concrete materials and structures is strongly influenced by cracks that are mostly induced by poor volume stability, and factors such as freeze–thawing, carbonization, alkali–aggregate reactions, chemical attacks, and rebar corrosion all affect durability [1]. The crack caused by the shrinkage of concrete remarkably increases permeability. Carbonation, chemical attacks, and reinforcement corrosion easily occur, and, as a result, the durability and service life of concrete structures are heavily deteriorated [2,3]. Obviously, quite a few kinds of deformation such as plastic deformation, thermal shrinkage, self-desiccation, drying shrinkage, and wet dilation are due to water loss and temperature variation [4,5,6]. In this way, we can conclude that the volume change of concrete results from internal stress that is induced by environmental or internal variations of temperature and moisture. In other words, the volume change of concrete is motivated by heat and moisture.

Moisture transport plays a main role in the durability and serviceability of cement-based materials [7,8]. It is generally believed that water transport in cement-based materials conforms to Fick’s law, which describes the molecular diffusion in porous media, such as cement-based materials [9,10]. However, some researchers think that molecular diffusion or ordinary diffusion occasionally occurs in concrete [8,11] and that Knudsen diffusion is not a negligible occurrence—rather it is one that results from the porous medium characteristics and pore distribution of cement-based materials. A theoretical formula for calculating the influence of the coefficient of Knudsen diffusion has been deduced on the basis of the theoretical equivalent diameter of mesco–micro pores, and the Knudsen diffusion mechanism must be considered in the kinetic theory of gas [11].

In real engineering structures, the deformation and cracking performance of concrete results from the coupling effect of environmental temperate, humidity, and structural loads. It is very important to investigate the hygro–thermo–mechanical deformation cracking performance and mechanisms of concrete. A number of studies have been published on the coupled hygro–thermo mechanical deformation of concrete, with hygro–thermal transference and induced deformation being investigated at high temperatures to analyze the behavior of concrete in fire [12,13,14]. Some studies based on thermodynamics of partially-saturated porous media will benefit the future research on the hygro–thermal–mechanical performance of structural concrete [12,15,16]. The deformations induced by temperature changes can be directly calculated by applying temperature loads to the nodes in the finite element analysis (FEA). On the contrary, it is hard to directly apply moisture loads to nodes with the current FEM approach. Considering that the current FEM approach does not allow for the direct application of moisture loads to nodes, like the approach can with temperature loads, a theoretical formula has been developed for calculating moisture-induced stress [17,18,19,20].

Similar to the effect of ambient temperature on the expansion of concrete samples, during the process of humidity exchange (moisture transfer), there is also a moisture gradient between the “layers” at different distances from the humidity exchange surface. Additionally, this gradient generates a stress differences under the mutual restraint between the layers. Therefore, by referring to the coefficient of thermal expansion (CTE) of materials, it is feasible to propose the concept of the coefficient of hygroscopic expansion (CHE) of cement-based materials. We believe that the application of the CHE to the FEA of calculating humidity effects will be beneficial in solving the difficult problem of calculation the deformation of humidity distribution. This coefficient should also be able simplify the calculation process of humidity deformation. 

At present, the CHE is mainly used to evaluate the moisture expansion and interfacial stress performance of ceramic tiles, clay blocks, and polymer composite materials [21,22]. It is a phenomenological evaluation index based on the final results of moisture absorption. The basic definition of the CHE can be defined as the volume (or linear) expansion rate under the moisture unit absorption rate of the material, which can be expressed by β. The β value is a fixed value within a certain range, and the expansion value can be calculated according to the value of β and the moisture absorption rate of the material.

According to the test methods of ceramic tiles—Part 10: Determination of Moisture Expansion (ISO 10545-10)—the initial length of the ceramic brick sample is measured twice (the second time is measured after 3 h) after being reheated and cooled, and then the sample is immersed in boiling water for 24 h. Afterwards, it is cooled down to room temperature, and its length is measured after 1 and 3 h to get the average value. The ratio of the difference between the two average values to the average initial length of the sample is the wet expansion of the ceramic brick. For polymer composites, the CHE (1/M^0^, where M^0^ is the moisture absorption rate unit) can be calculated as follows: One measures the change of expansion after the moisture absorption rate unit and then divides that change by the initial length of the sample to obtain the CHE.

The evaluation indexes of humidity in the air should include absolute humidity and relative humidity because analyses of cement-based materials and environmental humidity transmission are mainly based on moisture content. In addition, relative humidity is a function of temperature, so it needs further analysis and comparisons to select a benchmark index to evaluate the expansion coefficient of cement-based materials when the humidity unit changes. Moreover, the micro phase composition and proportion of cement paste also increase the complexity of determining the CHE. Therefore, the exact definition and determination method of the CHE of cement-based materials must be changed in order to achieve a more reasonable stress and deformation analysis through the direct application of moisture to the element node.

## 2. CHE of Cement-Based Materials

### 2.1. Definition of CHE

Similar to the definition of the CTE [23,24,25], the CHE can be defined as the volume change or length change caused by the change of the humidity unit inside a material; these changes can also be called the volume hygroscopic expansion coefficient (VCHE) and the linear hygroscopic expansion coefficient (LCHE), respectively. For convenience, relative humidity (φ) can be chosen to describe humidity or moisture. Its definition can be expressed as:

(1) $$\alpha_{sh}=\Delta V/\left(V\times \Delta \varphi \right)$$

(2) $$\beta_{sh}=\Delta L/\left(L\times \Delta \varphi \right)=\varepsilon_{sh}/\Delta \varphi$$

where *α_sh_* is the VCHE (με, equal to 10^−6^), *β*_sh_ is the LCHE (με), Δ*V* is the volume change of the specimen (m^3^), Δ*L* is the length change of the specimen (m), Δ*φ* is the relative humidity change (%), *V* is the volume of the specimen of material (m^3^), L is the length of the specimen of material (m), and ε_sh_ is the linear strain (με, equal to 10^−6^).

In 2006, Gawin et al. proposed that the shrinkage strain of concrete can be assumed to be directly proportional to the change of relative humidity through a phenomenological approach found in Professor Bazant’s research; this approach can be expressed as [26]:

(3) $$d\varepsilon_{sh}=\beta_{sh}\;d\varphi$$

where *d*ε_sh_ is the change of shrinkage strain (με) and *dφ* is the change of the relative humidity (%). Abbasnia et al. also considered that shrinkage, as one property of a material, can be described by an increment relation, which is expressed as the product of the shrinkage coefficient and the relative humidity of a capillary. This relation can be expressed as [27]:

(4) $$\varepsilon_{sh}=k_{sh}\times f\left(\varphi \right)$$

where *k_sh_* is the shrinkage coefficient (με) and *f(φ)* is the function of relative humidity of pores.

The above viewpoints and formulas confirm the feasibility and practicability of defining the wet expansion coefficient of concrete. 

It should be pointed out that the capillary tension theory and the Kelvin–Laplace formula are not applicable when the relative humidity is lower than 45%. Therefore, the application relative humidity (RH) range of the CHE proposed in this paper is 50%–100%.

### 2.2. Theoretical Derivation of CHE of Cement Based Materials

There is a static balance between the attraction and repulsion of pore water pressure to microstructures in hardened cement-based materials in the absence of self-drying or external drying. When drying occurs, the solution pressure in pores decreases due to the change of the capillary meniscus in the unsaturated pores, the static equilibrium is broken, the water vapor diffuses from the inside of the material to the surface, and more pores water is forced to evaporate to maintain equilibrium. It is generally believed that macropore water is lost first, and once the pore diameter reaches 50 nm, the capillary meniscus and micro shrinkage stress appear [28] and the internal relative humidity starts to decrease.

From the point of view of mechanical equilibrium, the relationship between pore pressure and meniscus curvature satisfies the Laplace equation [29,30]. The relationship between the negative pressure of pore fluid and the relative humidity in pores can be described by the Kelvin–Laplace equation, which can be expressed as [31,32,33]:

(5) $$p=\frac{-\ln\left(\varphi \right)}{v_{m}}RT$$

where *φ* is relative humidity (με), *R* is the universal gas constant (8.314 J·mol^−1^·K^−1^), T is the temperature in kelvins (K), *p* is the negative pore fluid pressure (Pa), and *v_m_* is the molar volume of water (m^3^·mol^−1^).

According to Mackenzie’s study, for a fully saturated porous medium, if the solid skeleton is linear elastic, the relationship between the change of mean hydrostatic pressure and the linear strain is as follows [29,33,34]:

(6) $$\varepsilon_{sh}=\frac{\Delta p\times S}{3}\left(\frac{1}{K}-\frac{1}{K_{0}}\right)$$

where Δ*p* is the average hydrostatic pressure change (Pa, which is caused by temperature change or humidity change), *S* is the saturation factor (the value of *S* can be considered equal to *φ*), *K* is the bulk modulus of the porous solid (GPa), and *K*_0_ is the bulk modulus of the solid skeleton of the material (GPa).

As is known, the aggregate accounts for a large part of concrete, and its volume is relatively stable. The shrinkage deformation of concrete mainly comes from the shrinkage of hardened cement paste, and when cement stone shrinks, aggregates limit the shrinkage of cement stone and then reduce the shrinkage effect of concrete. Therefore, the influence of aggregates and their force cannot be ignored in the analysis of the influence of average hydrostatic pressure (or pore hydraulic pressure) on the volume deformation of concrete [31,34,35]. Once a concrete mix proportion is determined, the amount (volume) of the concrete aggregate (coarse aggregate and fine aggregate) is fixed and remains stable in the development of concrete; the influence of aggregate on the linear strain of concrete can be considered by the volume fraction of the aggregate, *V*_a_*/V_c_*. The modified equation is:

(7) $$\varepsilon_{sh}=\frac{\Delta p\times S}{3}\left(\frac{1}{K}-\frac{1}{K_{0}}\right)\left(1-\frac{V_{a}}{V_{c}}\right)$$

where *V*_a_ is the volume of aggregates in concrete, *V_c_* is the volme of concrete (or cement mortar), *V*_a_*/V_c_* is the volume fraction of the aggregate in concrete (or cement mortar).

For partially saturated porous solids such as cement-based composites, the average hydrostatic pressure in pores can be replaced by pore fluid pressure. Thus, Δ*p* can be expressed as:

(8) $$\Delta p=p_{f}-p_{i}=\frac{-\ln(\varphi_{f})}{v_{m}}RT_{f}-\frac{-\ln(\varphi_{i})}{v_{m}}RT_{i}$$

where the subscript *i* indicates the start status and the subscript *f* indicates the end status.

Furthermore, the relation equation between humidity strain and the relative humidity of cement-based materials can be deduced and expressed as:

(9) $$\varepsilon_{sh}=\frac{R\times S}{3v_{m}}\left(\frac{1}{K}-\frac{1}{K_{0}}\right)\left(1-\frac{V_{a}}{V_{c}}\right)\left(T_{i}\ln(\varphi_{i})-T_{f}\ln(\varphi_{f})\right)$$

By dividing Equation (9) by Δ*φ*, the expression of the LCHE can be obtained theoretically.

(10) $$\beta_{sh}=\frac{R\times S}{3v_{m}}\left(\frac{1}{K}-\frac{1}{K_{0}}\right)\left(1-\frac{V_{a}}{V_{c}}\right)\frac{\left(T_{i}\ln(\varphi_{i})-T_{f}\ln(\varphi_{f})\right)}{\varphi_{i}-\varphi_{f}}$$

It can be seen that humidity causes the deformation effect of a cement composite. When the CHE, similar to the CTE, is used to describe the deformation effect, the relationship between the wet deformation, the dry deformation, and the LCHE is different from that between the thermal deformation and the LCTE, and its form is more complex. Even if the first three items’ expression of *β*_sh_ is constant, the LCHE is still not merely related to the relative change of relative humidity.

According to the above formula, for a certain saturated concrete material during a constant temperature drying process, when the parameter values are *K* = 25 GPa, *K*_0_ = 26 GPa [29,36], *V*_a_*/V_c_* = 0.65, S = 1, *T_i_* = *T_f_* = 20 °C, *φ_i_ =* 100%, and *φ_f_ =* 50%, the calculated LCHE is then:

$$\begin{array}{cc} \beta_{sh} & =\frac{R\times 50\%}{3v_{m}}\left(\frac{1}{K}-\frac{1}{K_{0}}\right)\left(1-\frac{V_{a}}{V_{c}}\right)\frac{\left(T_{i}\ln(\varphi_{i})-T_{f}\ln(\varphi_{f})\right)}{\varphi_{i}-\varphi_{f}}\\ & =\frac{8.314\times 0.5}{3\times 1.8\times 10^{-5}}\left(\frac{1}{25\times 10^{9}}-\frac{1}{26\times 10^{9}}\right)\left(1-0.65\right)\left(\frac{293.15\left(\ln1\right)-293.15\left(\ln0.5\right)}{1-0.5}\right)\\ & =16.8{\mu \varepsilon /\varphi }^{0} \end{array}$$

Here, φ^0^ means 1% change of relative humidity and με means 10^−6^.

By changing the value of *φ_f_* to 60%, 70%, 80%, and 90%, we can get the corresponding LCHE values of, respectively, 18.6, 20.2, 21.7, and 23με/φ^0^. Equation (10) is also applicable to cement mortar after changing the value of the volume fraction of the aggregate in mortar.

### 2.3. Rationality and Effectiveness of the Proposed CHE

Ayano and Wittmann proposed an empirical formula for the relationship between the dry shrinkage and relative humidity of mortar and concrete based on the moisture distribution and shrinkage test results of cement-based materials [37].

Mortar: *sh(h) =* 2170 × (1−*h*(t))^0.918^ (11)

Concrete: *sh(h) =* 1370 × (1−*h*(t))^0.749^ (12)

According to Equation (12), when the relative humidity is reduced from 100% to 50%, the drying shrinkage of concrete is 815.2 με. The corresponding LCHE can be deduced as 815.2/(100−50) = 16.3 με/φ^0^, which is very close to 16.8με/φ^0^ calculated according to Equation (10).

When the relative humidity of concrete drops to 90%, 80%, 70% and 60%, the LCHEs of concrete can be calculated as 24.4, 20.5, 18.5 and 17.2 με/φ^0^, respectively. These results are very close to those of Equation (10), as proposed in this paper. Therefore, it is feasible to calculate the LCHE by the proposed theoretical formula of Equation (10).

According to Equation (11), when the relative humidity is reduced from 100% to 50%, the drying shrinkage of mortar is 1148.5 με. The corresponding LCHE can be deduced as 1148.5/(100−50) = 22.97 με/φ^0^.

In the research of the hygro–thermo–chemo–mechanical modeling of concrete by Gawin et al. [26], the relationship between the stress induced by the change of humidity in concrete and the internal relative humidity was established according to the balance of pore pressure and mass conservation. Accordingly, the following relationships were established [26]:

(13) $$\varepsilon_{sh}=-\frac{\alpha }{3K_{T}}\left(\chi^{ws}p^{c}\right)I,\;\alpha =1-\frac{K_{T}}{K_{s}},\;p^{c}=-\rho^{w}\frac{RT}{M_{w}}\ln\left(\varphi \right)$$

where *α* is the Biot coefficient of concrete, *χ*^ws^ is the fraction of skeleton area in contact with water, *p^c^* is the equilibrium capillary pressure (Pa), *ρ*^w^ is the water density (kg·m^−3^), *K_s_* is the bulk modulus for solid phase (GPa, same as *K* above), *K_T_* is the bulk modulus for skeleton (GPa, same as *K_0_* above), *M_w_* is the molar mass of water (1.8 × 10^−3^ kg·mol^−1^), R is the universal gas constant (8.314 J·mol^−1^·K^−1^, *M_w_*/*ρ*^w^ equal to *v_m_* above), and **I** is the tensor unit of the second-order.

By substituting *α* and *p^c^* in the expression of ε_sh_ as expressed in Equation (13), ε_sh_ can be derived and expressed as:

(14) $$\varepsilon_{sh}=\frac{\left(K_{s}-K_{T}\right)\chi^{ws}}{3K_{T}K_{s}}\left(\frac{\rho^{{}_{w}}}{M_{w}}\times RT\times \ln\left(\varphi \right)\right)=\frac{RT\chi^{ws}}{3v_{m}}\left(\frac{1}{K_{T}}-\frac{1}{K_{s}}\right)\ln\left(\varphi \right)$$

Equation (14) is very similar to Equation (9), which was firstly derived and proposed by us. The differences are that the influence of the aggregate volume fraction (*V*_a_*/V_c_*) and the saturation factor (*S*) are considered in Equation (9), while the area fraction of the wetted solid skeleton (*χ*^ws^) is considered in Equation (14). Equation (14) describes the linear shrinkage strain of cement-based materials at a constant temperature.

Similar formal equations can also be derived by dividing Equation (14) by Δ*φ* (which equals *φ_f_* − *φ_i_*), which is consistent with the proposed theoretical calculation formula of the LCHE, as expressed in Equation (10). Therefore, Equation (14) also can be used to prove the rationality of the LCHE and its calculation method proposed in this paper.

The theoretical derivation method to determine the LCHE of cement-based materials has clear physical significance (see Equation (10)), but it is inconvenient to use this equation when the internal humidity field is hard to determine (relative humidity is also a function of temperature).

## 3. Determination of LCHE by Inversion Method

### 3.1. The Inversion Method

Besides the theoretical derivation, the LCHE also can be determined by a test. However, the measure of the internal humidity of materials is relatively complex. Moreover, it may take a long time to determine the deformation of a certain size of materials after the materials are completely balanced with different environments during testing, especially when the relative humidity of the environment is low. Therefore, the inversion analysis method can be considered for the determination of the LCHE of cement-based materials [38,39].

Here, the inversion analysis method mainly refers to a method in which the parameters required in the concrete simulation are not obtained by measurements in the actual field or laboratory; rather, they are obtained by using some ambient temperature and humidity or deformation measurement values to infer the relevant parameters, which are then applied to the whole simulation calculation process. When some parameters are difficult to obtain through testing or it is difficult to accurately reflect the actual situation, the inversion analysis is helpful for grasping the parameters that are needed for simulation and for more accurately calculating the physical and mechanical properties of concrete. In addition, this method can be used to calculate the required parameters in complex and unstable environments.

### 3.2. Drying Shrinkage Test

**Figure 1:**
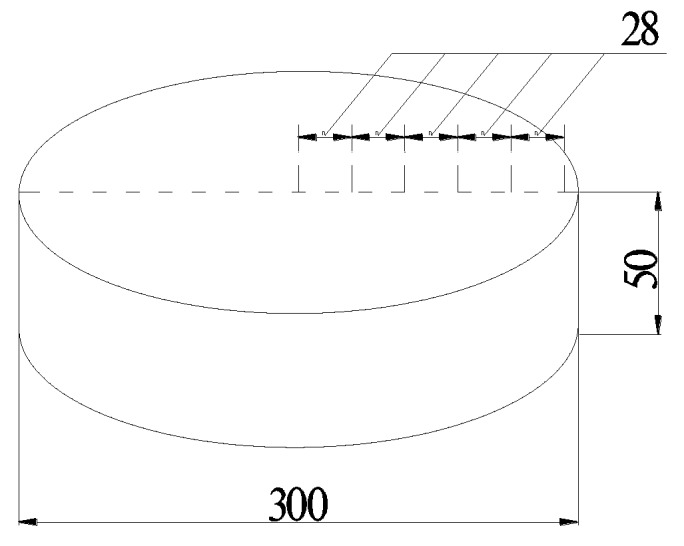
Schematic of the specimen size and steel needles distance.

For this paper, drying shrinkage tests were performed, and the distance change between steel needles was measured and analyzed. The cement mortar specimen was cylindrical disk with a height of 50 mm and diameter of 300 mm, and steel needles with different distances from the center (28, 56, 84, 112, and 140 mm) of the test piece were embedded on the upper surface so that the Vernier calipers could be used to measure the initial gauge distance and the distance change on different days. Considering of the symmetry, the probe of the steel needle was arranged with equal spacing along a certain radial direction, as can be seen in Figure 1. The mix proportion of the cement mortar specimen was cement: sand: water = 1:3:0.5. The cement was PO 42.5 Portland cement. The fine aggregate was all-purpose river sand. The compressive strength of hardened cement mortar cylinder specimen was 50 MPa.

The drying shrinkage experiment results were used to be compared with those from the drying shrinkage experiments to validate the constructed model and the obtained material properties.

### 3.3. The Finite Element Analysis

In inversion analysis, the finite element model, as the forward model, was created based on thermal–mechanical interaction with the transient analysis. The COMSOL Multiphysics software was used for the inversion analysis of the LCHE of cement-based materials.

#### 3.3.1. Finite Element Model

**Figure 2:**
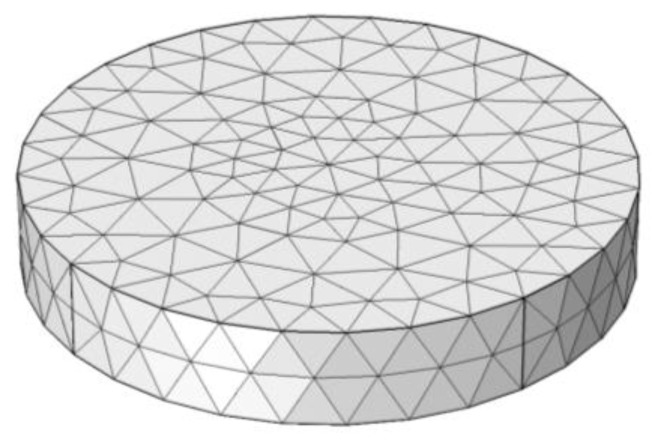
Free tetrahedral mesh of the specimen.

Because moisture distribution cannot be directly treated as moisture stress and deformation in COMSOL, a finite element model was created based on thermal-structural interaction with the transient analysis. In this coupled multi-physical model, the moisture diffusion in the mortar specimen was modeled as heat transfer, and the drying shrinkage behavior was modeled as thermal expansion. Thus, the moisture diffusion coefficient was defined as the thermal conductivity in the analysis. Based on that, the uncertain parameters of moisture expansion and moisture transfer on the concrete surface could be determined by inversion analysis. The model of specimen was meshed by using a tetrahedron element through the free tetrahedral method of COMSOL, as can be seen in Figure 2.

#### 3.3.2. Boundary Conditions

**Figure 3:**
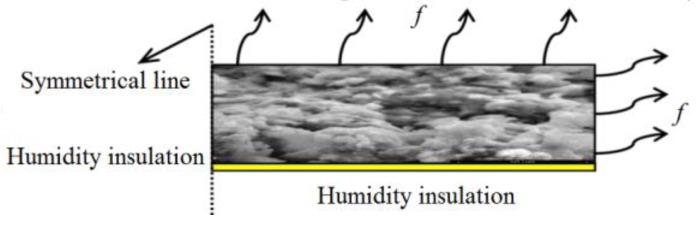
Sketch of boundary conditions in moisture diffusion model.

The mortar specimen was symmetrical, and the boundary conditions of that for moisture diffusion model are shown in Figure 3.

The boundary around symmetry axis was naturally assumed to be humidity insulation, and the bottom boundary was also approximately regarded as humidity insulation due to the fact that the moisture exchange coefficient of the epoxy coating on the bottom was very small. Ignoring the impact of water evaporation through the bottom side of the specimen was beneficial for the simplification of the analysis. The upper surface and the outer side of the circular specimen were assumed as the third kind of boundary condition (convection type), which is described by Equation (15).

(15) $$J=D_{mk}\times grad\left(\varphi \right)=f\times \left(\varphi_{s}-\varphi_{en}\right)$$

where J is the moisture flux (kg·m^−2^s^−1^), *φ*_s_ is the relative humidity on the surface (%), *φ*_en_ is the ambient relative humidity (%), *f* is the surface moisture transfer coefficient of mortar (SMTC in short, mm·day^−1^), and *D_mk_* is the diffusion coefficient of mortar (kg·m^−1^s^−1^).

The boundary conditions of the mortar specimen for mechanical model did not need to be defined because the specimen in the experiments was in the condition of free shrinkage.

#### 3.3.3. Initial Conditions

The cement mortar specimens were cured in a standard curing room for 28 days, and then they were put into a closed space with a relative humidity of 50%. Therefore, it was assumed that the internal relative humidity was 100%, that is the initial relative humidity was 100% when t = 0 on the first day of the test.

### 3.4. Inversion Analysis

In this inversion analysis, the finite element model established by COMSOL was a forward model. The optimization program was compiled according to the principle of least square method, and the interface with the optimization program code written by the MATLAB program was established. 

The Gauss–Newton algorithm was adopted in the optimization program. The LCHE and the surface moisture transfer coefficient were the two variables in the optimization code. The objective function can be defined as:

(16) $$F\left(\beta_{sh},f\right)={\sum_{j=1}^{m}\sum_{i=1}^{n}\left(E_{ij}-e_{ij}\right)}^{2}$$

where *E_ij_* is the *i*-th radial strain on the *j*-th day according to drying shrinkage test, *e_ij_* is the *i*-th radial strain on the *j*-th day according to the FEA results, *n* is the numbers of equidistant measuring points from the center of the specimen to the circumference of the surface in the test, and *m* is the drying duration of specimen in the experimental moisture and heat conditions.

The LCHE and the SMTC were assigned a value in each iteration cycle, and then the forward model was called and solved. Finally, this model was replaced with the objective function. When the objective function value was small enough, the finite element calculation results and experimental results were optimized, and then the inversion values of the LCHE and the surface moisture transfer coefficient were obtained.

**Figure 4:**
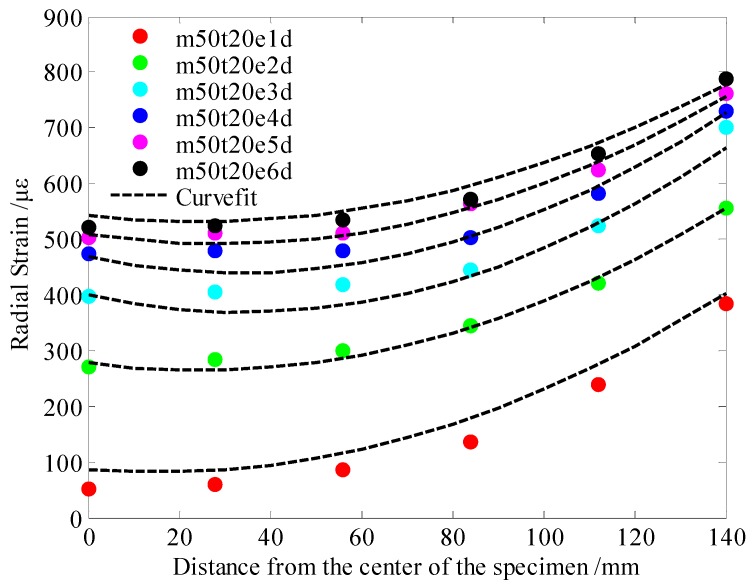
Experimental and optimal results of shrinkage at RH = 50%, T = 20 °C.

Based on the results of the drying shrinkage of cement mortar specimens at different ages under 50% relative humidity, the LCHE and the SMTC were estimated by the above inversion analysis process. The curve fitting results are shown in Figure 4.

**Figure 5:**
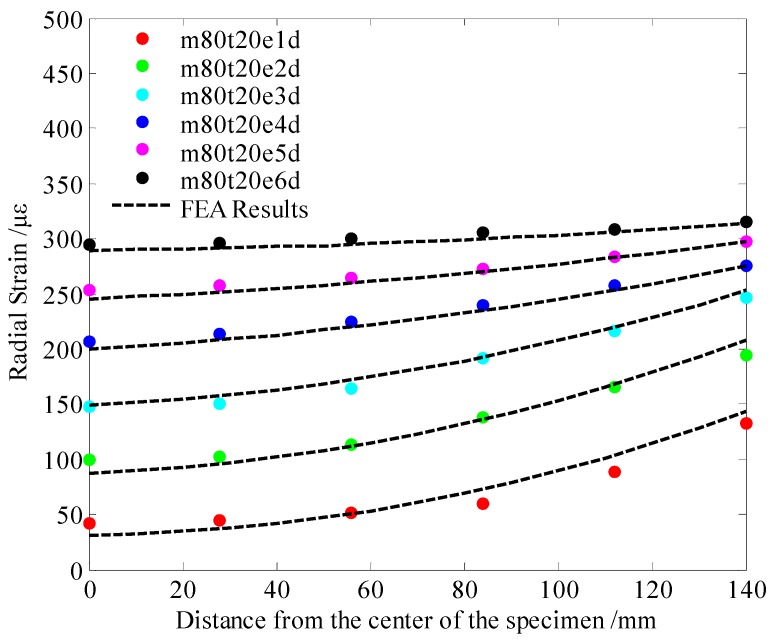
Experimental and FEA results of shrinkage at RH = 80%, T = 20 °C.

The LCHE of the cement mortar was 21.4 με/φ^0^, and the SMTC was 6.04 mm/d—these values were obtained by inversion analysis. The LCHE of 21.4 με/φ^0^ was close to that of 22.97 με/φ^0^, which calculated based on Equation (12), as proposed by Ayano and Wittmann [37]. As such, the LCHE and SMTC were used as the input parameters of the finite element model to predict the drying deformation of cement-based materials under different conditions. For example, this model was used to predict the shrinkage of cement mortars with the same specimen sizes and material parameters in an environment of 80% relative humidity and 20 °C. The experimental results and finite element simulation results are shown in Figure 5.

**Figure 6:**
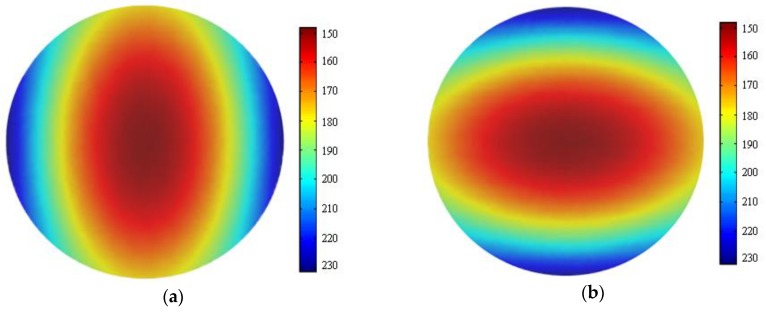

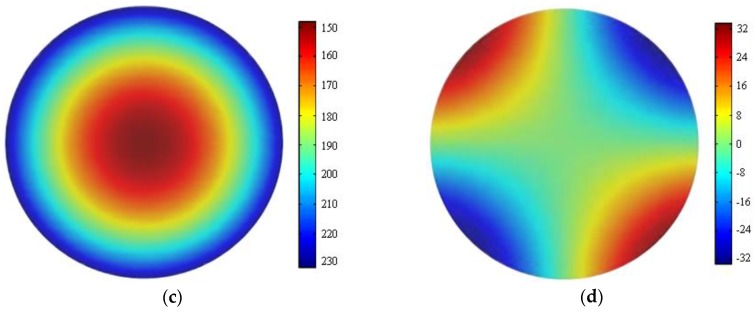
FEA results of the strain of the specimen at RH = 80%, T = 20 °C: (**a**) ε_x_; (**b**) ε_y_. (**c**) ε_r_; and (**d**) ε_xy_.

As shown in Figure 5, the results of FEA were very close to the experimental results, even if they were not always consistent. A strain nephogram of the FEA results of the internal strain of the specimen at the age of three days is shown in Figure 6.

## 4. Case Analysis on the Application of LCHE

### 4.1. Indoor Dry Shrinkage Test

**Figure 7:**
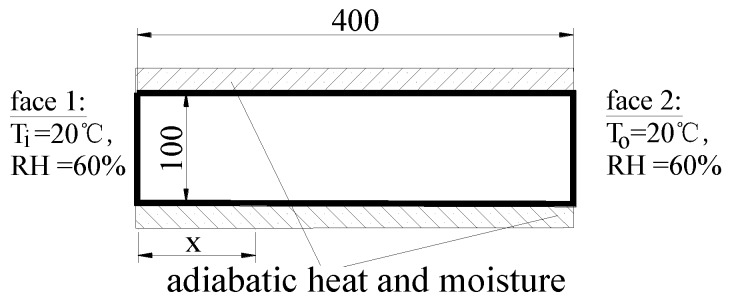
Schematic diagram of indoor dry shrinkage test specimen and conditions.

In the drying shrinkage test of concrete, the specimen size was 100 × 100 × 400 mm, the deformation probe was buried on the upper surface of the specimen, the standard curing was 3 d after formwork removal, and the specimen was placed in a constant temperature and humidity chamber (60 ± 5% RH, 20 ± 3 °C) for the length change and water loss test, as depicted in Figure 7. The concrete was prepared with PO42.5 ordinary Portland cement, pulverized fuel ash (PFA), natural coarse aggregates, natural fine aggregates, urban tap water, and polycarboxylate superplasticizer (1.0% wt of cement). The mix proportions of the concrete specimens are shown in Table 1. The properties of the three hardened cubic concrete groups (three specimen in one group) were tested, and the obtained values are listed in Table 2 [20].

**Figure 8:**
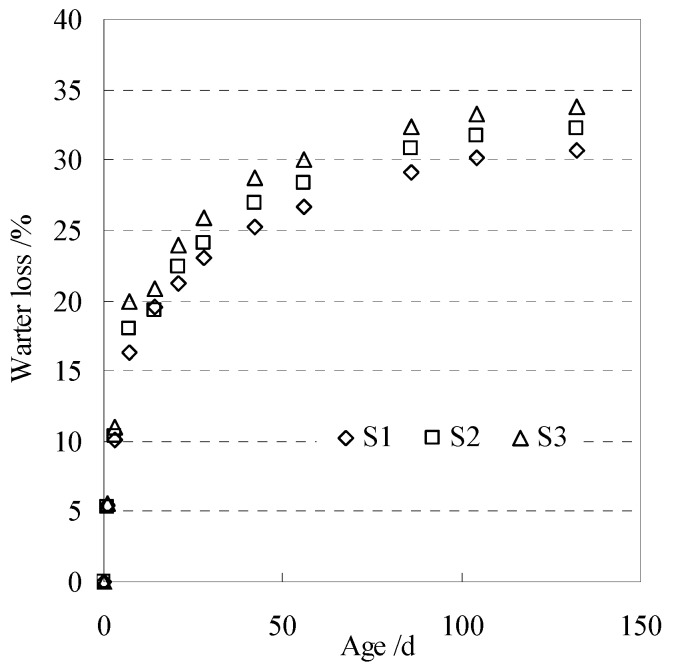
Measured water loss of concrete specimen.

The water loss and shrinkage of three groups of specimens (S1, S2, and S3) at different ages were measured and recorded for next analysis. The measured water loss rate of concrete is shown in Figure 8.

### 4.2. Internal Relative Humidity

**Figure 9:**
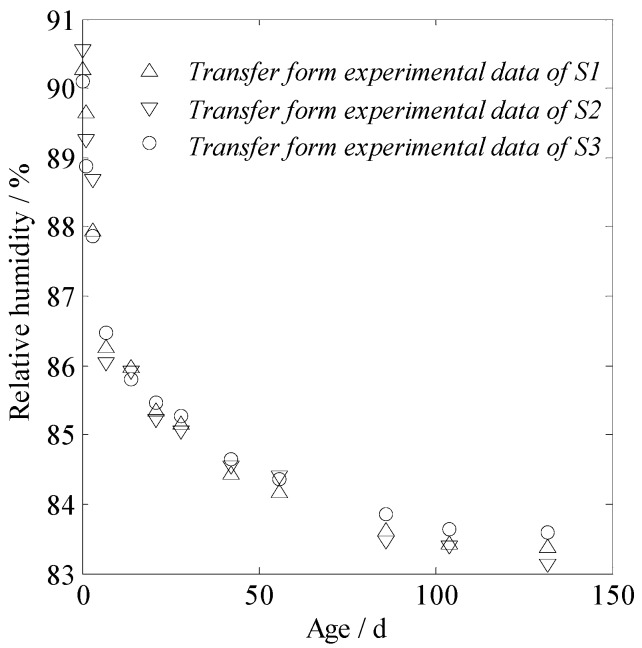
Internal relative humidity of concrete inferred from the measured water loss.

The internal relative humidity of the concrete was calculated according to the measured water loss rate of concrete, and these results are shown in Figure 9. The typical value of the LCHE, 16.8 με/φ^0^, as calculated with Equation (10), was used for concrete, and the shrinkage value was simply calculated according to Equation (2).

### 4.3. The Calculated and Measured Concrete Shrinkage

**Figure 10:**
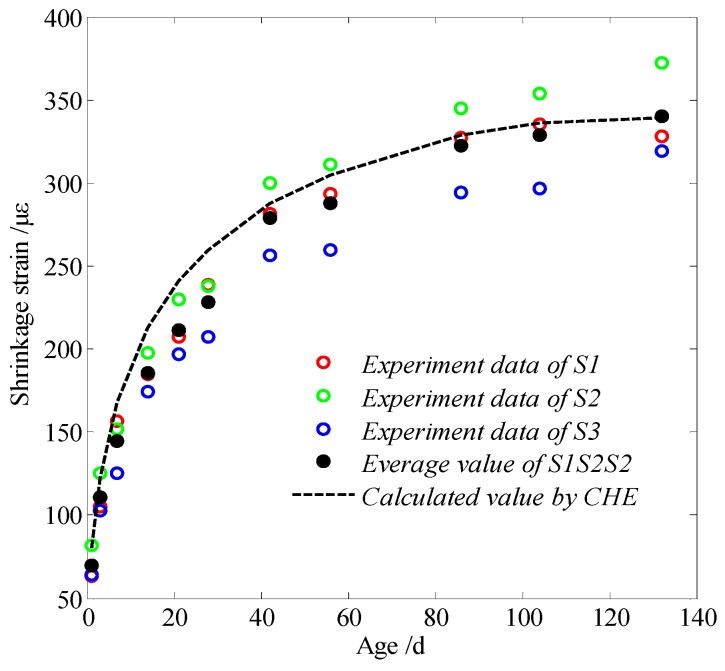
Shrinkage value and test results of concrete calculated according to the linear coefficient of hygroscopic expansion (LCHE).

The calculated value that was found by using the CHE and the measured concrete shrinkage value is shown in Figure 10.

The calculated value based on the LCHE in Figure 10 was very close to the average experimental values of three groups. Thus, it is feasible to calculate and predict the drying shrinkage of concrete according to the LCHE. However, in the situation where the calculated value in the early stage is higher than the test value, the later stage value tends to be the same, which results from the early hydration reaction of concrete. As such, the LCHE is mainly considered to be used for calculating the shrinkage that results from the change of the relative humidity of mature concrete. Therefore, there is a certain deviation when the LCHE of concrete is regarded as a fixed value to calculate the variation of concrete deformation.

The uncertainty of the experimental and calculating shrinkage of concrete can be analyzed by comparing the calculated values (CV in short) with the average experimental results (AER in short) at the same age; that is, the uncertainty can be found through the following: (CV – AER)/AER × 100%. For our experiments, the maximum, the minimum, and the average deviation were 16.1% (at the age of seven days), 0.2% (at the age of 132 days), and 8.8%, respectively.

## 5. Conclusions

In this article, the concept of the coefficient of hygroscopic expansion (CHE), which refers to the expression of the coefficient of thermal expansion (CTE), of cement-based materials is presented for the convenience of simulation calculation by using common FEM software and engineering applications.

The theoretical calculation formula of the linear coefficient of hygroscopic expansion (LCHE) of a cement-based material is deduced based on the Kelvin–Laplace equation and the Mackenzie Equation (which describes the relationship between the change of the mean hydrostatic pressure and the linear strain for fully saturated porous materials), as well as from the consideration of the volume fraction of the aggregate, the saturation factor, the average hydrostatic pressure change resulting from relative humidity variation in porous materials. Our obtained value of the LCHE of mortar and concrete that was calculated by the proposed formula was close to that in relevant references.

The theoretical derivation method to determine the LCHE of cement-based materials has clear physical significance, but it is inconvenient to use because it is sometimes difficult to determine the internal humidity field (relative humidity is also a function of temperature). The inversion method can be used to obtain the LCHE and the surface moisture transfer coefficient of a cement-based material. Thus, the necessary parameters can be obtained for the deformation simulation analysis of cement-based materials without a lot of tests. The experimental shrinkage of concrete is similar to that of FEA analysis because the moisture diffusion in a mortar specimen can be modelled as heat transfer in the COMSOL software, and this model can use the LCHE for inversion analysis.

A case analysis of the application of the LCHE was also conducted in this article for evaluating the applicable of the proposed CHE of concrete. The simulated results were close to experimental ones. Thus, it is feasible to calculate and predict the drying shrinkage of concrete through simple calculation by using the LCHE.

## Figures and Tables

**Table 1:** Mix proportions of concrete specimens (kg·m^−3^).

Groups	Cement	PFA	Sand	Gravel	water
S1	320	100	720	1180	140
S2	300	120	720	1180	140
S3	280	140	720	1180	140

**Table 2:** Properties of concrete.

Parameters	S1	S2	S3
Compressive strength (MPa)	45.8	45.5	44.1
Porosity (%)	9.6	10.2	9.8
Apparent density (kg·m^−3^)	2460	2475	2460
Saturated moisture content (%)	3.44	3.48	3.42
Thermal conductivity (W·m^−1^K^−1^)	3.01	2.99	3.05
Volume fraction of aggregate (%)	65	67	72
